# Disruptions and opportunities in sexual and reproductive health care: How COVID‐19 impacted service provision in three US states

**DOI:** 10.1363/psrh.12213

**Published:** 2022-11-09

**Authors:** Alicia VandeVusse, Philicia W. Castillo, Marielle Kirstein, Jennifer Mueller, Megan Kavanaugh

**Affiliations:** ^1^ Research Division Guttmacher Institute New York New York USA; ^2^ Research Division Formerly at Guttmacher Institute New York New York USA

## Abstract

**Context:**

The COVID‐19 pandemic abruptly disrupted the provision of sexual and reproductive health care in the United States.

**Methods:**

We conducted interviews with family planning clinic staff at 55 health care facilities in Arizona, Iowa, and Wisconsin in late 2020 and early 2021. We asked respondents about the challenges they faced and ways they adapted their service provision as a result of the pandemic. We conducted content and thematic analyses of the interview transcripts using an inductively developed qualitative coding scheme.

**Results:**

Family planning clinics and providers made a variety of changes to their clinic operations and service delivery. The three major areas of change for these facilities were implementation of COVID‐19 safety procedures, shifting service delivery and staffing to meet patient needs, and the rapid uptake and expansion of telehealth.

**Conclusion:**

While providers faced many challenges, they also described opportunities to innovate and rethink standard of care protocols that may continue to shape sexual and reproductive health care even after the pandemic abates.

## INTRODUCTION

The COVID‐19 pandemic disrupted the provision of health care throughout 2020 and 2021 in the United States (US). During the beginning stages of the pandemic, in early to mid‐2020, health care providers grappled with myriad impacts on their ability to provide care: resources were diverted to address the crisis,^1^ the health care workforce experienced unprecedented stress,^2^ patients avoided preventive health care out of fear of exposure to the virus,^3^ and health care facilities revamped workflows to provide services.^4^ These general disruptions to health care were compounded in several ways for sites providing family planning services,* such as federally qualified health centers (FQHCs), community health centers (CHCs), health departments, and specialized sexual and reproductive health (SRH) clinics. Many of these sites receive federal Title X funds, which support the provision of family planning services to people living on low‐incomes,^5^ un‐ and under‐insured individuals, and historically oppressed groups like Black and Latinx individuals and people of color. The pandemic occurred during a time of upheaval due to the Trump administration's 2019 Title X rule, which prohibited clinics from receiving Title X funding for family planning services if they provided abortion referrals.^6^ Furthermore, in the initial months of the pandemic, states issued emergency orders regarding essential services and these sites grappled with the burden of SRH services, particularly abortion, being deemed “non‐essential” by some states and localities.^7^, ^8^

Early in the pandemic, researchers began examining the effects of the COVID‐19 pandemic on the provision of SRH services. Many clinics had to temporarily close or cancel or postpone services, such as gynecologic care, contraceptive visits, and sexually transmitted infection (STI) testing.^9^ In early‐ to mid‐2020, Roberts and colleagues (2020) found that more than half of independent abortion clinics (clinics not affiliated with Planned Parenthood) canceled or postponed gynecologic services and contraceptive visits and 45% experienced disruptions to STI testing.^9^ Half of these clinics had staff who were unable to work due to the pandemic while navigating stress and confusion due to changes in clinic workflows.^9^ Maier and colleagues (2021)^7^ found severe reductions in SRH service provision during the first 6 months of the pandemic as fewer than half of states designated SRH care as essential and 14 states excluded abortion from the list of essential services. Of 61 contraceptive clinics across the US surveyed in April, August, and November 2020, over half experienced service interruptions at some point during their study period and 79% introduced or expanded telehealth for contraceptive counseling in response to the pandemic.^10^ Patients also reported canceling and delaying SRH care or having trouble receiving contraception due to the pandemic—notably an impact that has continued but become less prevalent as the pandemic has continued.^11^, ^12^

Research focusing on Title X clinics found that sites adopted practices to minimize risk of COVID‐19 exposure, such as requiring masks, closing waiting rooms, and limiting in‐person appointments while expanding telehealth infrastructure and care offerings.^13^ Providers adapted contraceptive service protocols to maintain patient access while reducing in‐clinic time through the use of curbside and mail delivery options.^13^ Timely innovations may reflect providers' experiences of adapting to disruptions from politically motivated service and funding restrictions.^14^

In this study, we build on this early research by exploring how family planning providers within and beyond the Title X program responded to the COVID‐19 pandemic. We extend prior work by collecting data in late 2020, after many early studies captured initial pandemic‐related impacts. The timing of our data collection allowed us to gather information about the range and persistence of pandemic‐related impacts. We conducted in‐depth interviews with staff providing SRH care at 55 clinics in three US states, including FQHCs, CHCs, hospitals, health department clinics, and specialized SRH clinics (sites where at least 50% of care provided is related to SRH, e.g., a Planned Parenthood facility). We investigated how these providers adapted their services and practices in response to COVID‐19. In addition, we delved into providers' perceptions of the benefits and challenges of utilizing telehealth during the pandemic. Understanding how SRH providers navigated the unprecedented upheaval of the pandemic, and sharing lessons learned, contributes to ensuring the continuation of high quality and timely care, despite disruptions within the health care system.

## METHODS

### Sample and recruitment

We conducted this study in Arizona, Iowa, and Wisconsin as part of the Reproductive Health Impact Study (RHIS), a multiyear (2017–2022) research and policy tracking initiative examining the impact of federal and state policy change on population‐, provider‐, and individual‐level outcomes related to publicly supported^†^ family planning care. The RHIS team selected states for their differing family planning policy contexts to allow for examination of state and federal policy impacts but without regard to the COVID‐19 situation in each state (see RHIS website^15^ for more information on the overall study). Of the study states, only Wisconsin designated some SRH services essential,^7^ although Arizona and Iowa enacted state policies to expand telehealth access while Wisconsin did not.^16^ As one component of the RHIS initiative, we conducted in‐depth interviews with clinic administrators, family planning managers, or staff members in similar roles at family planning clinics. Although this study was not originally designed to examine the impact of the pandemic, we expanded our research focus and interview guide to explore this issue.

Aligned with the focus of the larger RHIS work, we identified our sample through referral from the states' current and/or former state‐level Title X grantee organizations administering this funding within their networks, snowball sampling from interviewees, and outreach to eligible respondents whose facility participated in other components of the RHIS initiative. Our study team stopped recruiting participants when we reached data saturation,^17^, ^18^ demonstrated by respondents reporting information represented in previous interviews. Between August 2020 and January 2021, we conducted interviews with 57 respondents at 55 family planning health care facilities (at two sites, we spoke with more than one respondent, per respondents' request).

### Data collection

Each interviewer (PC, MK, and JM) conducted one pilot interview with a respondent meeting our eligibility criteria in a non‐study state (Maine), to avoid piloting with potential respondents. The study team used feedback from the pilot interviewees to improve the flow and clarity of the interview guide before beginning the study.

Within the existing interview guide, focused on understanding the broader impact of policy change on the delivery of publicly‐supported family planning care, we developed a section to gauge the effect of the COVID‐19 pandemic on this care. At the outset of the interview, we asked respondents to provide background information on their health facility, including the SRH services they provide and changes that occurred at the larger health care network in the 18–24 months before the interview. Next, interviewers asked respondents questions about how their facility had been impacted by the pandemic. Specifically, we asked interviewees about any changes to clinic operations, contraceptive service provision, financial well‐being, and how social distancing measures (such as telehealth and curbside service) were or were not implemented as well as the subsequent reactions from providers, staff, and patients. For the purposes of this research, we define telehealth as the provision of health care services by a health care provider via technology, including video calls, phone calls, and app‐based services.^‡^ Interviewers also asked respondents to speak to the potential permanence of any changes made due to the pandemic.

Three members of the study team (PC, MK, and JM) conducted interviews, which lasted approximately 75 min. We offered Zoom video, Zoom audio, and phone interview options to respondents to minimize disruptions to clinic staff schedules and to maximize the ability to speak with providers across states. We audio recorded interviews and interviewers and participants were in private spaces during the interview. All participants orally consented prior to the interview; participants could stop participating in the interview at any time or decline to answer any interview question. We offered respondents a USD75 gift card as remuneration for participation. The Guttmacher Institute's federally registered institutional review board approved the study.

### Data management and analysis

A third‐party transcription service transcribed audio recordings of interviews and five research assistants reviewed transcriptions for accuracy and removed identifying information. We used NVivo12 to organize and code deidentified transcripts and generate code reports. We inductively developed a coding scheme based on the interview guide and existing literature and conducted content and thematic analyses of the respondents' narratives after reading all transcripts. Initially, we divided eight transcripts among the analysis team (PC, MK, JM, and AV) for independent coding. The team met to resolve differences and strengthen the coding scheme by developing new codes. The analysis team then divided up all transcripts and at least one team member coded each transcript using the refined coding scheme. The analysis team met regularly to review coding progress and resolve analysis questions. Once the coding process was complete, we generated code reports for facilities in each state by clinic type. We divided code reports among team members and reviewed them to explore sub‐themes before summarizing findings and sub‐themes into matrices, organized by state and clinic type. The analysis team conducted multiple rounds of review to identify and consolidate themes. In the following section, we describe how the COVID‐19 pandemic affected sites across clinic type and state.

## RESULTS

The 57 participants were from 17 sites in Arizona, 20 sites in Iowa, and 18 sites in Wisconsin (see Table 1), a roughly even split by state. Respondents were typically clinic managers or family planning coordinators, although specific roles and titles vary across sites. Over one‐third of respondents worked at specialized SRH clinics, while over one‐quarter were employed at a CHC or an FQHC. Just under 25% worked at health department sites and the remaining 14% worked at hospital sites or other clinic types.

**TABLE 1:** Number and percentage of respondents by state and site type (*N* = 55)

Characteristic	Arizona (*n* = 17)		Iowa (n = 20)		Wisconsin (*n* = 18)		Total (*N* = 55)	
	*n*	%	*n*	%	*n*	%	*n*	%
Specialized sexual and reproductive health site	5	29	7	35	8	44	20	36
Community health center/Federally qualified health center	6	35	5	25	4	22	15	27
Health department	5	29	3	15	4	22	12	22
Hospital	1	6	2	10	1	6	4	7
Other	0	0	3	15	1	6	4	7

*Note*: Percentages may not sum to 100 due to rounding.

As a result of the pandemic, family planning clinics and providers across all states and facility types made a variety of changes to their clinic operations and service delivery. Our findings are organized according to three domains that emerged among our respondents' accounts: (1) implementation of safety procedures in clinics to minimize the risk of exposure to COVID‐19; (2) shifting service delivery and staffing to meet pandemic‐related patient and provider needs; and (3) experiences with telehealth, including benefits and challenges of telehealth adoption or expansion. We identify the clinic type and state when using direct quotes from respondents and, where relevant, describe differential experiences within these domains.

### Implementation of safety procedures

Respondents reported making several changes to decrease their patients' and providers' risk of exposure to COVID‐19 while continuing to provide care. Many facilities implemented screening procedures by deploying facility staff to ask patients whether they had any symptoms associated with COVID‐19 before entering the facility and taking their temperatures upon arrival. If a patient was found to have COVID‐19 symptoms, they were rescheduled to avoid potential exposure and clinic shutdowns.

I think the big thing is really, just, you know, screening people when they make appointments and when they come in, so we know they are safe. […] we've had a couple of cases where somebody came into the clinic and we realized that they were a potential exposure, and we had to shut down a room.—Hospital site, Iowa.

Facilities also increased the use of personal protective equipment (PPE) such as requiring, and often providing, masks for patients, staff, and providers as well as requiring gloves, eye protection, and gowns for providers. Some facilities made these changes to prioritize patient safety in the absence of specific government regulations. For example, respondents in Iowa and Wisconsin described implementing masking protocols when their state did not have an applicable mask mandate. Some facilities experienced shortages of supplies making routine use of PPE a challenge. In some cases, staff re‐used items or paused clinic services due to insufficient access to PPE.

Simultaneously, staff increased the frequency and depth of cleanings throughout clinics and between each patient, particularly for “high touch” areas. This often placed an increased burden on staff and contributed to some sites offering fewer appointments. These reductions were often short‐term and abated as clinics adjusted to the new protocols.

We've had to greatly, greatly limit our hours and then even on top of the hours, how many patients we can see in a day because we've spaced them so far apart so we never have more than one patient in the waiting room at a time. We have to clean and wipe down the waiting room, the restroom and the exam room in between every single patient. We're really only able to see maybe 7 or 8 patients a day spacing that far apart. Every single day is booked and even then there's patients that can't get in. So the huge limitation in services is how we're affected.—Health department site, Arizona.

Many clinics changed their hours or altered their appointment practices to modify, reduce, or eliminate walk‐in services and waiting areas to accommodate social distancing and avoid crowds. Respondents described how their clinics' elimination or significant reduction of walk‐in appointments and waiting rooms allowed staff to control the number of patients in the space at any given time. In lieu of a waiting room, some patients were asked to wait outside or in their cars until their appointment time.

We have stopped walk‐ins. We used to have a lot more walk‐ins where people would say, “Do you have time to do a pregnancy test? Can I get in today?” Something like that. We make them do appointments.—Health department site, Iowa.

Similarly, many clinics that previously allowed patient guests for comfort, convenience, or support grappled with whether and when to allow guests in the face of social distancing requirements and fears of viral exposure. Respondents described a range of policies that generally required patients to be unaccompanied unless certain circumstances applied, such as parents/guardians of small children or adolescent patients who wanted a parent/guardian present.

We do prefer that they come by themselves. Sometimes that doesn't happen, especially with single moms with littles at home. They oftentimes will have to bring their children, but that's completely understandable. We just make accommodations for that.—Health department site, Iowa.

Some clinics also allowed a guest for certain procedures, such as prenatal ultrasounds. In general, however, clinics maintained no‐guest policies to limit the number of people entering the clinic.

### Shifting service delivery and staffing

Beyond changing clinic operations, clinics altered their service delivery protocols to minimize the risk of COVID‐19 and shifted staffing to accommodate evolving patient and provider needs linked to the pandemic. Many clinics saw a decrease of in‐person visits as a result of revamped clinic protocols, postponement of non‐urgent appointments, and decreased patient demand for in‐person care, although several specialized SRH sites reported increased patient volume due to their nearby health care counterparts suspending SRH care to focus on COVID‐19 response. Facilities also expanded telehealth offerings and adopted curbside and mailing options for certain contraceptive refills (e.g., pill, patch, ring, emergency contraception) and STI testing and treatment.

Many respondents reported that their clinics canceled or postponed appointments deemed non‐urgent, such as elective procedures, “well woman” exams, and pap smears, during the initial months of the COVID‐19 crisis, when less was known about how the virus spreads and treatments and vaccines were not yet available. As a representative from a specialized SRH in Arizona stated, “I feel that people were scared and are still scared and it's holding people back from getting their reproductive care that they need; getting their birth control on time.”

Postponements were mostly temporary and allowed clinics to protect staff and patients while developing new workflows around telehealth. A few respondents, particularly those at health department sites, reported that their facilities had not adopted telehealth or only had phone‐appointment capabilities, mainly due to lack of infrastructure and staffing. Still, many clinics rapidly initiated or increased their telehealth offerings to provide services that were limited by the pandemic and challenging to provide in person. Fewer in‐person visits meant that many sites experienced a decrease in revenue.

However, the federal emergency authorization allowing Medicaid to reimburse providers for telehealth visits equivalently to in‐person care^19^ facilitated increased revenue from expanded telehealth services. Several respondents described how use of telehealth shifted as the pandemic continued. At the outset, some clinics routed all patients through an initial telehealth visit to determine if in‐person care was warranted.

We had a pretty decent number of months there where visits were only scheduled in‐patient if they had had a telemedicine visit first, and then it gets sussed out that we had certain things that could be scheduled in person.—Hospital site, Wisconsin.

Over time, as the most acute concerns regarding COVID‐19 gradually abated, many clinics developed systems for determining if telehealth was necessary prior to an in‐person visit, and some began allowing patients to select their mode of care. This meant “conversation‐based” care such as assessments and consultations occurred via telephone or video calls. Many of our respondents described providing contraceptive pill refills, pill prescription extensions, STI screenings, and presumptive STI treatments over telehealth for the first time. Sites also used telehealth to take patient history, counsel on safe sex practices, and, where applicable, to provide medication abortion follow up.

When possible, some sites also expanded contraceptive access by prescribing or providing more cycles of oral contraceptives or lengthening the time between depot‐medroxyprogesterone acetate (DMPA) shots. In addition, many of our respondents provided needed medications, methods, or supplies through pharmacies, curbside service, mail delivery, or quick pick‐up at a designated place in the clinic to minimize the amount of time spent in the same room as patients.

Anything that we wouldn't need to get them in for, or if they want to limit the amount of time they are in a building, we can do everything that we need to that way [telehealth], and then maybe just have them come in and just get the exam, or just leave a urine sample or something like that, or receive their medication.—Other site, Wisconsin.

Sites also had to contend with shifts in staffing capabilities and needs, as changing workflows affected staffing models, COVID‐19 exposure and quarantines led to unpredictability in staff availability, and financial concerns led to staff cuts at some clinics. Cuts in staffing also affected the number of appointments clinics had available for SRH services. Staff burnout was a prevalent concern for most sites but was especially pronounced at health departments and FQHC sites, where staff were often engaged directly in the local COVID‐19 response.

I've observed what I would say is a lot of burnout with the staff. […] I just think people feel stretched in between performing their normal job duties and then also having to do other things that are asked of them that interfere often with their ability to care for the people they're supposed to be caring for.—Health department site, Arizona

### Telehealth benefits and challenges

One of the major changes reported by almost all of respondents was the dramatic uptake or expansion of telehealth offerings for family planning services and other SRH care. This dramatic shift in care modalities has many benefits, such as avoiding COVID‐19 exposure and reducing access barriers. However, it also brought serious challenges, some of which were unique to the circumstances under which telehealth was adopted and some of which are related to telehealth more generally.

Our respondents noted many benefits of telehealth for SRH care provision. First and foremost, telehealth allowed patients and providers to avoid unnecessary exposure to COVID‐19. Particularly during the initial months of the pandemic when little was known about COVID‐19, this was a major benefit for both staff and patients, though it remained an important way to avoid exposure as the pandemic continued. As a representative from a specialized SRH site in Wisconsin reported, “… at first [it was like] oh I'm not sure if I like this, but at the same time we're realizing that this is the best, safest, efficient way to provide care.”

Respondents also noted that staff benefitted from having the ability to work from home, which only became possible through telehealth. Another benefit many respondents described was added control over patient flow in the clinic. Because patients typically received in‐person appointments after a telehealth evaluation, the clinic staff were better able to prepare for patient needs and anticipate the number of patients to be seen in a day.

Many respondents lauded the ways telehealth reduced barriers to access for patients, such as transportation, childcare, time off work, or geographic distance from the clinic. Telehealth provided convenience to patients, allowing them to access care from home, work, or other locations, as needed. Removing these barriers provided access to care for patients who might otherwise forgo or postpone receiving SRH care.

The clinicians are happy to do it and still be able to help the patients and limit exposure to the virus itself and the patients say exactly the same thing, as far as their work schedule or children's schedule—having that access through the televisit has been super helpful.—CHC site, Arizona.

Several of the challenges respondents described witnessing in their clinics relate to the unique circumstances under which telehealth was adopted, namely with little pre‐existing infrastructure, time constraints, and amidst the stress of the pandemic. Those challenges have largely abated as time has gone on and clinics have adjusted.

One early challenge was the lack of pre‐existing infrastructure for telehealth in many family planning care settings. Since telehealth was not widely available in most clinics prior to the pandemic, some staff were unfamiliar with the technology, and clinics did not have telehealth protocols in place. This sometimes led to confusion about how to schedule patients, particularly when scheduling was handled by call centers outside of facilities.

Then, of course, we had to figure out how to do the flow of those visits differently than when the person was in front of you. So, that was a challenge, because we were doing a lot more of it on the phone, in advance, and then we would call them and tell them to get in the queue for the provider so that we had to rework the whole patient flow as far as the clinic. I really think a lot of those visits took more time than if they had been in the clinic.—Hospital site, Iowa.

Furthermore, because many respondents reported feelings of urgency to initiate telehealth services and continue providing patient care, telehealth was adopted rapidly. This sometimes resulted in chaotic rollouts or the selection of sub‐optimal programs or workflows that had to be readjusted over time. Finally, many respondents noted that the general stress experienced by staff and patients during the early months of the pandemic coincided with the adoption of telehealth. As a representative from a CHC site in Arizona reported, “My eyes were tired from looking at a video. My ears. I was exhausted by hearing, and it's not natural to communicate with a delay. Physically, we were all very tired.” The challenges associated with the rapid uptake of telehealth were largely resolved with time. Staff grew more familiar with telehealth programs and software, workflows were developed and adapted to appropriately route patients to in‐person or telehealth appointments, and the changes to clinic operations mentioned above have mitigated staff concerns about COVID‐19 exposure in clinic.

On the other hand, some of the telehealth challenges noted by respondents are associated with this modality of care more generally, such as internet access issues, privacy concerns, loss of in‐person connection, and continuity of care concerns. These challenges have proven more difficult to address. One of the more intractable issues reported by respondents was patients' lack of consistent, reliable access to the technologies needed for telehealth, such as internet, phone service, and video capabilities. As a representative from a CHC site in Iowa reported, “[A challenge of telehealth is] patients not having Wi‐Fi or not having access to a computer, or having a smart phone that only has so many minutes that run out.”

Although many respondents noted that patient access to smartphones is widespread, they frequently described issues connecting with patients for telehealth appointments, because patients may have limited minutes for phone use, data for internet access, or text messages. These issues are compounded when patients lack access to consistent Wi‐Fi service in their places of residence or have literacy or language barriers. Coordinating interpreters during telehealth appointments can also be a challenge.

Because we noticed that the appointment that the patient has not been connected you know, or has not initiated the connection when she or he needed to be connected, and we were wondering why. So, we have to call the patient, and he goes, well, I have no idea what the text says because the text is in English. And even if it will be in Spanish, they would still be like… some of our folks will have a hard time trying to understand it because unfortunately not everyone reads and writes Spanish.—Specialized SRH site, Wisconsin.

Another common telehealth challenge raised by our respondents was the loss of the ability to ensure consistent access to a confidential space for patients during appointments. This caused privacy concerns, particularly when discussing intimate topics, such as contraception, sexual health, and intimate partner violence. Respondents also mentioned fear that this could introduce Health Insurance Portability and Accountability Act (HIPAA) violation issues.

…The biggest thing is, patients are not always able to have access to a safe, confidential, secure place to do the visit, because access to Wi‐Fi is not universal. Sometimes, I think we assume that it is, but it's not. If you're going to use free Wi‐Fi, it's usually connected to a public place. I've had people have to do their visit in a bathroom because they were at a coffee shop and they needed to use the Wi‐Fi, but that was the only secure place that they could do it.—Specialized SRH site, Wisconsin.

The barriers to and challenges of telehealth were not exclusive to patient experiences. Many respondents mentioned that clinicians found aspects of providing care more difficult through telehealth. These difficulties related to rapport‐building, reading non‐verbal communication, and having in‐depth conversations and connecting with patients on a personal level, such as those required for contraceptive counseling.

…I have to have that personal touch with a patient. I don't feel we could give them that personal touch that they need, because the population that we work in, there's a lot of patients that don't know a lot of things, or how to take their birth control. I feel we're giving them… We're not helping to the way that we can, versus when they're in here and they're more comfortable and they ask us questions.—Specialized SRH site, Arizona.

Another telehealth‐related issue raised by many respondents was continuity of care for patients needing in‐person follow‐up care. Many providers described a desire to continue offering telehealth services after concerns of COVID‐19 exposure subside. However, most noted that this was conditional on the continuation of telehealth being reimbursed by Medicaid and private insurance similarly to in‐person care as outlined in emergency legislation enacted during the COVID‐19 pandemic.^20^, ^21^ The dramatic expansion of telehealth services was largely facilitated by this legislation, and if reimbursement rates decrease, clinics may reduce or eliminate telehealth offerings to maintain their financial health.

That's a bit of a challenge with telehealth is insurance paying. Pretty soon they're going to stop paying for audio only telehealth, they will only pay for tele video calls. Some of our patients don't have smart phones, so they can't participate in that. So, it is a barrier.—CHC, Arizona.

## DISCUSSION

The first year of the COVID‐19 pandemic created many challenges for the provision of SRH care and our respondents described a variety of adaptations their clinics made to keep providing needed care while still protecting patients and staff. Similar to Weigel and colleagues' findings among obstetrician‐gynecologists (Ob/Gyns), our respondents reported experiencing major pandemic‐related challenges and changing service delivery to meet patient and provider needs as a result, particularly through the rapid expansion of telehealth.^4^ We found that changes were made while clinics dealt with concerns about decreased revenue, workflow adaptations to telehealth platforms, shifting patient needs, and staff burnout. While staff morale was a concern for most of our respondents, we found that this concern was more prevalent among respondents from FQHCs and health departments than other site types, mirroring Prasad and colleagues' findings that health care workers' experiences of COVID‐19‐related stress varied by worker role.^2^

Overall, respondents described the ways in which the disruptions of the pandemic and policy changes promoted innovation and rethinking of protocols that may carry through even as the immediate concerns of the pandemic abate. The innovations in access that providers experimented with during the pandemic, such as extending contraceptive refills without in‐person appointments, lengthening the time between DMPA shots, and providing presumptive STI treatment should be codified into practice guidelines to ensure patient access is at the forefront.^22^, ^23^ Prescription requirements for contraceptive methods that have been widely studied and used should be reassessed to allow for greater access outside of the clinical space and reduce barriers for those who do not have easy access to a physician.^24^ Providing over‐the‐counter access, along with mail and curbside pickup to safe, effective and acceptable contraceptives can reduce inequities in contraceptive access.^24^ Furthermore, officially recognizing SRH care as an essential service, as 14 states did in early 2020, may help to ensure that patients can access vital health care and that providers have the resources they need to continue serving patients.^7^, ^22^ Although we did not find differential results by state, despite different state responses to the pandemic, this should not be taken as evidence that state policy does not matter, especially given that prior research in these study states found that patients in Arizona were more likely to report difficulties accessing SRH care than those in Iowa and Wisconsin.^25^ However, this research project was designed in the context of the overall Reproductive Health Impact Study rather than to shed light on COVID‐19 responses and all three of the states enacted some form of pandemic response: Wisconsin included SRH in the definition of essential services^7^ and Arizona and Iowa expanded telehealth services.^16^ This may explain why state differences were not pronounced in our qualitative findings.

Although we found minor differences in COVID‐19 impact among our respondents based on site type, mentioned above, most of our findings were similar across site types, demonstrating that many SRH clinics experienced similar challenges in the early months of the pandemic and implemented similar procedures as a result. We uncovered fewer disruptions to care due to COVID‐19 than Roberts and colleagues' study of independent abortion providers, perhaps in large part because contraception was not targeted by politicians seeking to restrict access to the extent that abortion was in the early months of the pandemic.^7^ In addition, some specialized SRH clinics and CHCs/FQHCs in our sample are part of larger networks of clinics, which allows for greater resource sharing, and this may further mitigate the impacts as compared to those of Roberts and colleagues. However, in the wake of the Supreme Court's decision in *Dobbs v. Jackson Women's Health Organization*, there has been increased concern regarding the ripple effects and potential for subsequent attacks on access to contraception,^26^ which further solidifies the need for recognition of sexual and reproductive health care as essential, time sensitive services for which access must be preserved and codified.

Our work extends prior research by allowing for a fuller examination of the experience of initiating and expanding telehealth offerings and the benefits and challenges brought about therewith. Similar to Weigel and colleagues' findings among Ob/Gyns, our respondents reported dramatic increases in their use of telehealth to provide SRH care.^4^ New adoption of telehealth prevented unnecessary exposure to COVID‐19 and provided convenience to patients by reducing many of the barriers that stymie health care access generally, such as transportation, childcare, work obligations, and geographic distance.^11^ However, research has found that contraceptive patients rank their experiences with telehealth services as less patient‐centered than in‐person care, which highlights the need for providers to continue to improve their telehealth services.^27^ Several challenges our respondents expressed mirrored those reported elsewhere, such as the limitations of telehealth for provision of certain services and the difficulties experienced by some patients in using telehealth.^4^ Our study expands this literature by distinguishing between the telehealth issues that stem from the stressful and rapid conditions under which telehealth was expanded and which have been ameliorated as the pandemic wore on and those that require more innovation and attention to address. These latter challenges, such as ensuring patient access to Wi‐Fi for appointments, assisting providers in building rapport remotely, and ensuring continuity of care for patients moving between in‐person and telehealth care modalities should be top priorities for policy makers and health care providers as they work to codify telehealth as a viable service modality.

For telehealth to support health care equity, structural changes must be made to ensure all patients have access to necessary technologies. While health inequities are a multi‐pronged systemic issue, determining ways to further reduce barriers to telehealth may be particularly important for Black and Latinx individuals, people of color, and people living on low‐incomes who use publicly supported health facilities for their health needs.^4^, ^5^ These groups have been more likely to experience delays in accessing SRH services during the pandemic^12^ and, due to existing systemic inequities, have been disproportionately affected by COVID‐19.^25^, ^28^ Furthermore, telehealth allows increased access to health care for rural, geographically dispersed populations. These barriers often preclude patients from receiving needed care, and the removal of these barriers provides access for patients who otherwise might forgo or postpone receiving health care. However, telehealth workflows must continue to be improved to ensure that its use reduces barriers to care rather than introduces different ones. Health care systems must ensure telehealth platforms, as well as explanatory materials and trainings, are available in patients' languages, and they must reduce literacy barriers to access. Telehealth offers the possibility of expanding access to interpreters, but coordinated efforts are required to ensure availability. Providers should develop scripts for communicating to patients the privacy needs for their appointment, and they should undergo training on how to enhance rapport and ensure the patient‐provider relationship does not suffer through telehealth use. Finally, health systems must agree on standards of care for telehealth to ensure that health care quality is maintained or enhanced through the use of telehealth.

For the promise of telehealth to be achieved, it must also be supported by insurance providers and the government.^27^ This includes coverage of the provision of tools for patients to engage in telehealth effectively (e.g. blood pressure monitors and scales) as well as support for proper health care provider reimbursement. The expansion of telehealth in family planning clinics was facilitated by many states' Medicaid programs adjusting payment rates and expanding covered services for telehealth during the pandemic,^29^, ^30^ and it is essential that sustainable reimbursement rates continue, even after the pandemic subsides, as a means of increasing access.

This study provides a deeper understanding of how SRH care providers have been impacted by the COVID‐19 pandemic and how they have adapted to continue serving patients. Through in‐depth interviews with respondents from clinics of different types in three states, this study demonstrates that providers implemented safety procedures to minimize COVID‐19 exposure, changed service delivery protocols, and expanded telehealth use. However, these results may not be generalizable to health care settings beyond SRH clinics or geographic locales, although as described above our findings align in some key ways with studies of Ob/Gyns, abortion providers, and other health care providers. Furthermore, our results represent the perspectives of certain staff at the participating clinics, rather than those of their patients, so their understanding of patient needs should be explored with patients themselves in future work. Another potential limitation is nonresponse bias, as non‐participating clinics may have experienced different pandemic‐related impacts than those who participated.

## CONCLUSION

Our respondents reported many challenges and adaptations to care provision in the face of the COVID‐19 pandemic, which demonstrated sexual and reproductive health care providers' resilience, ability to adapt, and their commitment to providing health care services to their communities safely. Providers implemented a variety of safety procedures to minimize COVID‐19 exposure while continuing to offer care, and they developed new methods of service delivery such as curbside and telehealth. By sharing SRH providers' challenges and innovations during COVID‐19, this study offers insight into how to ensure the continuation of high quality and timely care despite unprecedented disruptions.

## Endnotes

* Family planning services include, but are not limited to, contraception services, pregnancy testing and counseling, fertility and infertility services, preconception health services and sexually transmitted infection services. For the purposes of this analysis, we do not include abortion care in our definition of family planning services.

^†^
A publicly supported clinic is a site that offers contraceptive services to the general public and uses public funds (e.g., federal, state or local funding through programs such as Title X, Medicaid or the FQHC program) to provide free or reduced‐fee services to at least some patients.

^‡^
In our interviews, respondents use the terms telemedicine and telehealth interchangeably.
